# Post-metabolic response to passive normobaric hypoxic exposure in sedendary overweight males: a pilot study

**DOI:** 10.1186/1743-7075-9-103

**Published:** 2012-11-16

**Authors:** Chad Workman, Fabien A Basset

**Affiliations:** 1 School of Human Kinetics and Recreation, Memorial University of Newfoundland, St. John’s, NL, A1C 5S7, Canada

**Keywords:** Metabolic rate, Indirect calorimetry, Substrate partitioning, Hypoxia

## Abstract

**Background:**

The present pilot study was designed to test the impact of passive acute normobaric hypoxic exposure (PAH) and passive short-term normobaric hypoxic exposure (PSH) conditions on energy expenditure (EE) and substrates utilisation (glucose and lipid oxidation).

**Methods:**

Eleven participants have completed the PAH session while the control group (CG) underwent a simulated experimental condition in normobaric normoxic condition. A subset of 6 participants underwent an additional six 3-hour sessions on consecutive days. Metabolic rates were obtained pre- and post-treatments on the morning following an overnight (12 hours) fast in PAH, PSH, and CG groups.

**Results:**

The statistical outcomes showed a significant increase in EE for PAH, control, and PSH while a shift in substrate utilization towards lipid sources was only detected for PAH and PSH, respectively.

**Conclusion:**

This pilot study showed that passive acute normobaric hypoxic exposure did affect EE and fuel utilization in sedentary overweight males and that further passive normobaric hypoxic exposures (PSH) magnified these metabolic adjustments. These outcomes provide valuable information for further research in the area of hypoxia as a new therapeutic strategy to improve the management of weight loss.

## Background

Obesity seriously threatens the public health in the westernized world [1]. Even though efforts have been made to reduce obesity, efficient solutions proposed from biological and behavioural sciences often do not appeal to all members of society as an effective means to reduce body fat. Obesity impairs physical performance and leads to an unfitness status that negatively affects whole body metabolism and daily energy expenditure [2].

Even though many scientific reports confirm the beneficial effects of regular physical activity on reduced mortality from all causes, including obesity, a mix of personal (e.g., past experience with exercise, health status), behavioural (e.g., skills), and environmental (e.g., access to facilities, type of program) factors influence both uptake and maintenance of exercise. In fact, most of the obese population in North America has a sedentary lifestyle, and approximately 60% of individuals who initiate an exercise program drop out within 3 to 6 months, well before any significant health benefits [3].

New perspectives have, however, emerged from studies on human hypoxic tolerance showing that some effects might be potentially beneficial in specific physiologic or pathologic conditions and could be an effective means to reduce body fat [4]. In fact, according to the most recent studies on the topic, moderately obese subjects did significantly lose weight after intermittent hypoxic exposures [5]. Hypoxia has also been associated with an augmented metabolic rate and an increase in energy expenditure [6], a general metabolic perturbation that might affect fuel utilization [7]. Previous studies have reported that the majority of weight loss in lean fit subjects was attributed to fat mass reduction, possibly due to increased fat oxidation [8-10].

Yet, what effect low O_2_ concentration has on post-hypoxic substrate metabolism is still not fully understood. One can postulate that hypoxia exposure triggers metabolic responses similar to, but not identical with, exercise-induced metabolic disruption [11]. If so, post hypoxic exposures (acute and short-term) might result in shifting substrate utilization towards lipid sources, due to the greater dependency on glucose under hypoxia [12]; a metabolic pattern that slightly differ from the excess post-exercise oxygen consumption concept and its related mechanisms [13].

To date, however, no study has examined the effect of passive acute and short-term hypoxic exposure on post-metabolic responses as related to substrate partitioning and energy expenditure. The present pilot study was, therefore, designed to test the impact of acute and short-term normobaric hypoxic exposure on substrate partitioning and energy expenditure. It was hypothesized that (a) acute normobaric hypoxic exposure would shift the fuel utilization towards lipid sources and would increase basal metabolic rate; (b) short-term normobaric hypoxic exposure would result in a cumulative effect on the above-mentioned metabolic responses.

## Methods

### Participants

Eleven sedentary overweight males – (BMI: 28±5 kg·m^-2^; height: 179±8 cm; weight: 88±5 kg) aged 28±3 years old – participated in this study after having a medical examination. In addition, four sedentary overweight males – (BMI: 27±2 kg·m^-2^; height: 173±12 cm; weight: 82.2±11.5 kg) aged 36±5 years old – served as a control group. Participants were recruited from student population on Memorial University campus in response to advertisements posted on campus. They all filled in a Physical Activity Readiness Questionnaire (PAR-Q) to determine level of activity, and to screen for a history of any risk factor and health condition including smoking history, hypertension, cardio-respiratory disease, diabetes, musculoskeletal injuries or family history of any of the above-mentioned conditions in addition to known previous mountain sickness or altitude symptoms. They were excluded from the study if they took prescribed medication of any kind, were smokers or diagnosed as having; respiratory problems, heart disease, hypertension, chronic or acute illness, anxiety disorders, and drug or alcohol abuse. They were also excluded from the study if they were involved in any form of sports or structured exercise programs during the previous 12 months. The selected participants, then, attended an orientation session in which they were given information about the equipment and the experimental design, in addition to undergoing anthropometrics measurement. Finally, each participant signed a written informed consent in compliance with the declaration of Helsinki and with Memorial University’s ethics committee regulations. Participants received one hundred dollar in compensation for their time and commitment as approved by the Human Investigation Committee.

### Experimental design

Three sets of data were obtained from which two distinct groups underwent either a passive acute normobaric hypoxic exposure (PAH) consisting of a 3-hour normobaric hypoxic exposure or a passive short-term normobaric hypoxic exposure (PSH) consisting of 7 days of a single 3-hour normobaric hypoxic exposure session. The third set of data was obtained from the control group (CG). Participants were unaware of control or experimental conditions. Basal and post-treatment metabolic rates were measured on the first (day 1) and last day (day 7) of the experiment. During the treatment the oxygen concentration was maintained ~ 80% blood O_2_ saturation (S_p_O_2_) as monitored by pulse oximetry. Food intake and physical activity were obtained from daily logs to estimate the total daily energy expenditure. All experimental sessions were conducted at the same time of the day. Eleven participants have completed PAH session while CG (n = 4) underwent a simulated experimental condition in normobaric normoxic condition. A subset of 6 participants (BMI: 26±7 kg·m^-2^; height: 177±9 cm; weight: 83±12 kg) underwent an additional six 3-hour sessions (PSH) on consecutive days.

#### Metabolic rate determination

Participants were first subjected to a basal metabolic rate (BMR) and were, therefore, requested to comply with the following criteria prior to undergoing BMR: (1) to engage in no exercise during the preceding 36-hours; (2) to ingest no caffeine or alcohol during the preceding 24-hours; (3) to consume a last meal before 20:00 on the preceding evening and drink only water afterwards; (4) to travel to the laboratory by car or public transportation; (5) to rest for 30-min on a bed in a quiet environment prior to commencing recording metabolic data. Upon arrival to the laboratory, participants were placed in a comfortable, supine position in a quiet environment in preparation for metabolic rate measurement via indirect calorimetry technique. The participants were instructed to remain quiet and relaxed during data collection, but to stay awake. Upon the completion of treatment (experimental and control), participants underwent anew a metabolic rate measurement following the procedures described above.

#### Experimental condition

A modified generator, equipped with a semi permeable filtration membrane (nitrogen filter technique), continuously pumping air at a flow rate of 20 l·min^-1^ into a facial mask lowered atmospheric O_2_ concentration to expose participants to an isometabolic stress, that is, the same relative S_p_O_2_ (~80%) during treatments (GO_2_ Altitude, Biomedtech, Melbourne, Australia). Gas concentrations were monitored by oxygen sensor (Cambridge Sensotec, Cambs, UK). S_p_O_2_ and heart rate (HR) were recorded online with a pulse oximeter (GO_2_ Altitude, Biomedtech, Melbourne, Australia). In addition, blood pressure and acute mountain sickness inventory questionnaire were collected every 30-min over the course of exposure. During treatment and control sessions participants were allowed to perform sedentary tasks such as reading, writing, or television viewing. The session was stopped if blood pressure rose more than 30 systolic points from baseline, heart rate increase or decrease of more than 20 beats per minute from baseline or an increase in AMS score above 3 points.

#### Cardio-respiratory measurements

Oxygen uptake ($\dot{V}O_{2},1\cdot \min^{-1}$), carbon dioxide output ($\dot{V}CO_{2},1\cdot \min^{-1}$), breathing frequency (B_f_) and tidal volume (V_T_) were continuously collected with an automated breath-by-breath system (Sensor Medics® version Vmax ST 1.0) using a nafion filter tube and a turbine flow meter (opto-electric). Minute ventilation and respiratory exchange ratio were calculated from B_f_ and V_T_, and from $\dot{V}O_{2}$ and, $\dot{V}CO_{2}$ respectively. Heart rate values were transmitted with a Polar heart rate monitor (PolarElectro, Kempele, Finland). The signal was transmitted to and recorded via the metabolic cart. Prior to testing, gas analyzers and volume were calibrated with medically certified calibration gases (15% O_2_ and 5% CO_2_) and with a 3-liter calibration syringe, respectively. In addition, to insure accurate calibration of the cart, the propane gas calibration was performed to assess the sensitivity of the oxygen and carbon dioxide analysers.

#### Fuel selection

Oxidation rates (g·min^-1^) of carbohydrate (CHO_ox_) and lipid (FAT_ox_) were calculated according to the following equations [14]:

(1) $$\begin{array}{ccc} CHO_{\mathrm{ox}}\left(g\min^{-1}\right) & = & 4.59\dot{V}CO_{2}\left(L\min^{-1}\right)\\ & & -3.23\dot{V}O_{2}\left(L\min^{-1}\right) \end{array}$$

(2) $$\begin{array}{ccc} FAT_{\mathrm{OX}}\left(g\min^{-1}\right) & = & 1.70\dot{V}CO_{2}\left(L\min^{-1}\right)\\ & & +1.70\dot{V}O_{2}\left(L\min^{-1}\right) \end{array}$$

where $\dot{V}O_{2}$(l·min^-1^) and $\dot{V}CO_{2}$(l·min^-1^) were corrected for the volumes of O_2_ and CO_2_ corresponding to protein oxidation (1.010 and 0.843 l·g^-1^, respectively). For relative contribution of substrates protein oxidation rate was estimated at 66 mg·min^-1^ based on previously published urinary urea excretion measurements made on 12-h post-absorptive men with normal CHO reserves [15,16].

Atmospheric conditions (atmospheric pressure, humidity, and temperature) were collected over the course of the study.

### Data reduction and analyses

Pre- and post-exposure metabolic rates were truncated by 10-min out of 30-min of data collection. The procedure discarded the first and last 5-min in order to nullify any metabolic rate fluctuation due to familiarisation with the facemask and the expected termination of data collection. The remaining 20-min segment was, then, integrated, normalized over time, converted in and expressed as energy expenditure (EE) in kilocalories (Kcal) and as oxidation of glucose and fat (mg·min^-1^). The same truncation and integration were applied to HR, S_p_O_2_ pre- and post-exposure as well as during exposure.

### Statistical analyses

All data are presented as mean and standard deviation unless otherwise specified. First, owing to unequal group sizes, paired t-tests were performed separately on PAH and CG to detect any significant change in EE and substrate partitioning. In addition, paired t-test was run on S_p_O_2_ to insure that an isometabolic stress was applied on PAH during treatment. Second, a two-way ANOVA [2 periods (Pre and Post) x 6 epochs (30, 60, 90, 120, 150, and 180-min)] with repeated measures on HR, and BP was computed to assess the effect of PAH. Third, a two-way ANOVA [2 periods (Pre and Post) x 2 time (day 1 and day 7)] with repeated measures on EE and substrate partitioning was run to detect the effect of PSH, and on S_p_O_2_ as for the previous condition. Finally, a three-way ANOVA [2 periods (Pre and Post) x 6 epochs (30, 60, 90, 120, 150, and 180-min) x 2 time (day 1 and day 7)] with repeated measures on HR, and BP was computed to assess effect of treatment. Prior to running the statistical plans data sets were verified for normality (Wilk-Shapiro, Lilliefors, and Kolmogorov-Smirnov tests). As well, the assumption of sphericity was tested. When statistical significance was reached (alpha level of *p*≤0.05), *post*-*hoc* analyses were run to identify where significant mean differences occurred. The statistical Package for Social Sciences (SPSS, version 19) was used for all statistical analyses (SPSS Inc., Chicago, USA).

## Results

### Environmental parameters

Room temperature was maintained between 22 and 24°C throughout the experiment, while atmospheric conditions averaged between 99.5±0.6 kPa and 86.0±10.7 Rh% at 7:30 and 99.6±0.5 kPa and 73.4±15.0 at 12:30 for barometric pressure and humidity, respectively.

### Health related parameters

Based on the visual inspection of the acute mountain sickness score no statistical analysis was run. AMS scores ranged from 0 to 1 – with a minimum possible score of 0 and a maximum of 15 – confirming that participants did not experience any symptom of AMS. In addition, no participant has been removed from the experiment based on the criteria set by the experimenters such as blood pressure rising more than 30 systolic points from baseline, and/or a change of more than 20 beats·min^-1^ in heart rate or an increase in AMS score above 3 points.

Although participants were required to record a daily diet and physical activity log, data obtained were insufficient for further analyses. However, from the questionnaire, the self-reported physical activity level of all applicants was below the Canadian guidelines for the general population.

### Cardiovascular parameters

Descriptive statistics for S_p_O_2_, HR, and BP (systolic and diastolic) are presented in Table 1. None of the above-listed parameters did reach significance neither in PAH nor in PSH. As expected S_p_O_2_ did not vary much – because it was monitored and controlled during treatment – as reflected by the coefficient of variation [CV = 3.5%, 3%, and 2.9%] in PAH and PSH, respectively. Although a large within-condition variability (on average: SD ± 11.2) was observed due to individual biological variance, the average HR in PAH and PSH did not vary much – by 6% at most. The systolic and diastolic pressures were affected neither in PAH nor in PSH, the greatest variation reaching only 3% and 4% for SBP and DBP, respectively. For CG the average HR was 72 ± 7; unfortunately, the S_p_O_2_ and BP were not recorded for this group.

**Table 1:** Cardiorespiratory parameters for PAH and PSH

**PAH (n = 11)**			**PSH (n = 6)**			
S_p_O_2_ (%)	81 ± 2		82 ± 2		82 ± 2	
	3-hour exposure		Day 1		Day 7	
	Pre	Post	Pre	Post	Pre	Post
HR (beat·min^-1^)	76 ± 7	83 ± 6	81 ± 6	89 ± 6	80 ± 5	85 ± 11
SBP (mmHg)	116 ± 8	116 ± 9	114 ± 9	117 ± 10	114 ± 9	113 ± 8
DBP (mmHg)	80 ± 8	78 ± 8	80 ± 9	80 ± 9	76 ± 9	79 ± 6

Mean ± SD.

### Energy expenditure

**Figure 1:**
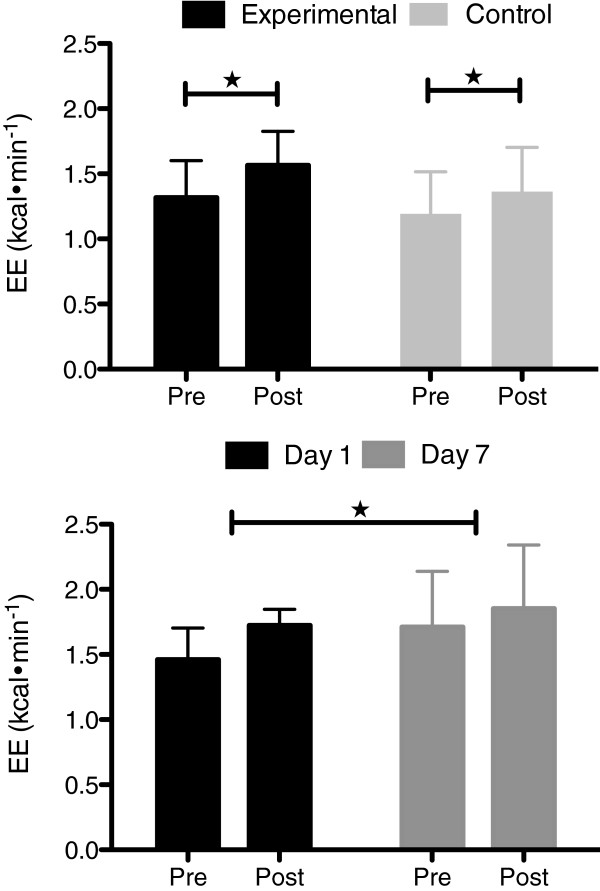
**Energy expenditure (EE) in Kcal·min**^**-1**^**pre- and post-treatments for PAH (n = 11) and control (n = 4) [upper panel], and pre- and post-treatments in day 1 and day 7, respectively for PSH (n = 6) [lower panel].** ★ Significantly different from pre- to post-treatments, and from day 1 to day 7.

The Figure 1 depicts the pre- to post-treatment variation in EE (PAH, CG, and PSH groups on the upper and lower panel, respectively). The paired *t*-test outcomes revealed that EE significantly increased by 16% (*p* = 0.002) and by 12.5% (*p* = 0.029) for PAH and CG, respectively. The analysis of variance revealed that EE for PSH significantly differed from day 1 to day 7 increasing by 12% (*p* = 0.037). However, it is worth noting that EE did increase by 18% on day 1 and continued to increase by 3% on day 7.

### Fuel utilization

**Figure 2:**
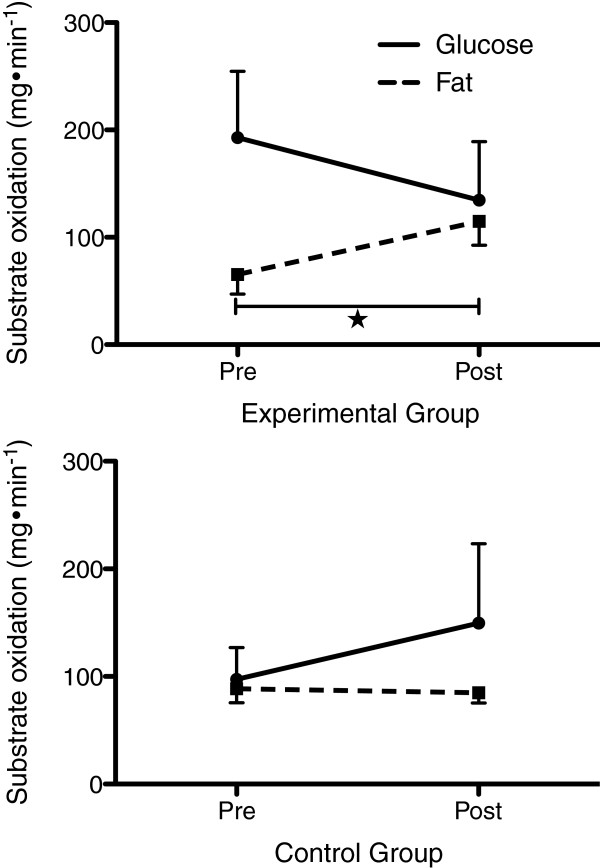
**Substrate oxidation (glucose and fat) in mg·min**^**-1**^**pre- and post-treatments for PAH (n = 11) [upper panel] and control (n = 4) [lower panel], respectively.** ★ Significantly different from pre- to post-treatments.

**Figure 3:**
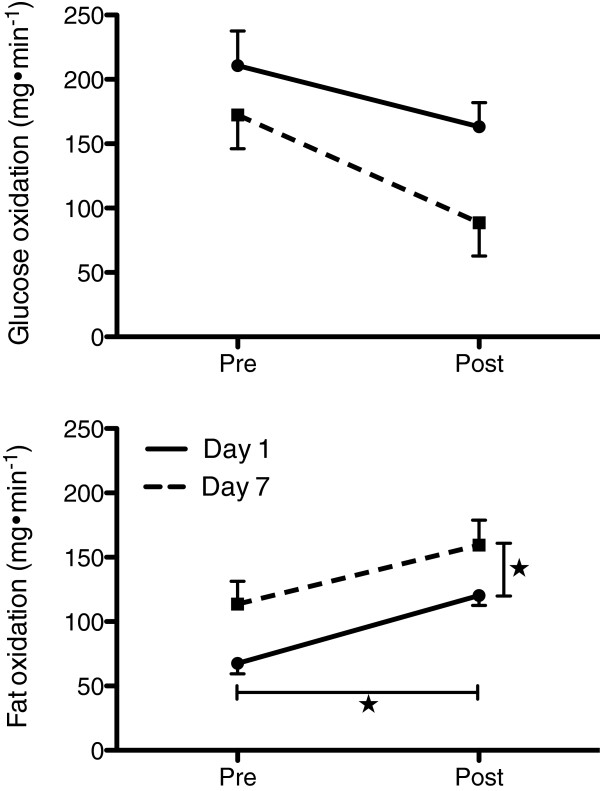
**Fat oxidation [upper panel] and glucose oxidation [lower panel] in mg·min**^**-1**^**pre- and post-treatments in day 1 and day 7, respectively for PSH (n = 6).** ★ Significantly different from pre- to post-treatments, and from day 1 to day 7

Figure 2 depicts the substrate oxidation for PAH and CG, respectively. From the upper panel one can see that glucose oxidation significantly decreased by 31% (*p* = 0.034) and fat oxidation significantly increased by 44% (*p* = 0.001) from pre- to post-exposure. In comparison on lower panel CG increased glucose oxidation and slightly decreased fat oxidation by 35% and by 4%, respectively but none of those scores did significantly differ from each other. Figure 3 (upper panel) showed that in PSH glucose oxidation, although not statistically significant, decreased from pre- to post-exposure by 34% (*p* = 0.057), both days combined. Glucose oxidation did decrease from pre- to post-exposure on day 1 by 26%, and continued to decrease from pre- to post-exposure on day 7 by 49%. In parallel and as displayed on Figure 3 (lower panel), fat oxidation significantly increased by 44% (*p* = 0.006) from pre- to post-exposure and by 29% (*p* = 0.05) from day 1 to day 7.

## Discussion

The purpose of this pilot study was to examine the effect of acute and short-term normobaric hypoxic exposure on post-metabolic responses. The novelty of the study resides in its experimental design. In fact, to the authors’ best knowledge, it is the first time that a study was designed to record post-metabolic responses in normoxia immediately after being exposed to acute and short-term passive hypoxia. As postulated the outcomes showed a shift in substrate partitioning and an increase in energy expenditure in both acute and short-term conditions. Indeed, acute and short-term normobaric hypoxic exposures did lead to an increase in lipid oxidation and a decrease in glucose oxidation along with an augmented basal metabolic rate. The main outcomes of the investigation confirmed prior research reporting short-term hypoxic exposures to be of sufficient amplitude to initiate positive adaptive responses [4,17].

### Cardiovascular parameters and health population characteristics

As mentioned by Lippl *et al*. [18] most published studies have involved fit individuals or normal-weight participants rendering the interpretation quite difficult since confounding variable such as exercise, fitness and cold exposure might have interfered with altitude / hypoxic exposure. It has also been pointed out that acclimatory responses to hypoxia depend on the inter-individual variability, and training profile [19]. For instance, in highly trained athletes the extent to which performance improves could be too little to reach statistical significance [20] owing to optimization of their physiological systems (e.g., respiratory, cardiovascular, muscular). The data obtained, therefore, could barely be applied to unfit / sedentary obese subjects. In the current study, caution was made on selecting individuals with a BMI > 25 kg·m^-2^ who were not involved in any form of sports or structured exercise program during the previous 12 months. Monitoring these parameters strengthened the effect of hypoxic exposure. In addition, the participants were submitted to a isometabolic stress, that is, S_p_O_2_, was monitor and controlled during treatment to maintain oxygen desaturation around 82% at 2% of the initial target (80%). The rationale underlying the isometabolic stress was to standardize as much as possible the environmental stimuli between subjects and to minimize the inter-subject variability. This may explain why none of the participants experienced acute mountain sickness event. No cardiovascular parameter was affected by the treatment neither in acute or short-term exposure. However, although none significant, heart rate was higher post- compared to pre-exposure values, a result that matched the higher significant EE observed in the present study as a consequence of the hypoxic treatment. It sounds then logical to observe an augmented cardiopulmonary function along with an increase metabolic rate since the cardiopulmonary system delivers the oxygen and substrate for replenishing stores [13].

### Energy expenditure

Relatively brief periods of hypoxic exposure via a hypobaric chamber or inhalation of a normobaric hypoxic gas mixture stimulates erythropoietin [21] resulting in improved reticulocyte count, haemoglobin, and, haematocrit. These studies suggest that there is a good correlation between circulatory O_2_ transport and higher basal metabolic rate. Some authors have reported a significant increase in energy expenditure at least transiently at altitude or under hypobaric / normobaric hypoxia. For instance, Butterfield *et al*. [22] reported a significantly elevated BMR above sea level at the initial stage of a 21-day altitude stay whereas Mawson *et al*. [23] showed an increase in resting metabolic rate after 3 days of a 12-day altitude stay. Lastly, Lippl *et al*. [18] did show a significant increase in BMR after an altitude stay (~2650 m) in sedentary obese people. Contrary to previous studies on the topic, the authors carefully controlled for the confounding factors, such as cold and physical activity. In the present study all groups did significantly increase EE from pre- to post-hypoxic exposure at rest and in a thermo-neutral condition. This result was partly explained by the transition from supine to seated position. However, there was a greater increase in EE in PAH compared to CG (16% vs. 12.5%) in addition to a carry-over effect from day one to day seven in PSH confirming that acute and short-term hypoxic exposure have had a greater effect than energy requirement of the seated position. The cumulative effect observed over the course of 7 days (12.5% increase) corroborated previous studies showing an increase in basal metabolic rate following altitude stay [18,23-26]. The mechanisms behind the increase in EE at altitude / hypoxic exposure still remain debated. However, Mawson *et al*. [23] and Louis *et al*. [27] have suggested that an increase in sympathetic nervous system activity might play a role.

### Fuel utilisation

The main outcome of this pilot study showed that hypoxic exposures (acute and short-term) did affect substrate partitioning. The rationale underlying the current study was, based on the mechanisms contributing to the elevated post-exercise metabolism, that hypoxic exposure results in a general metabolic perturbation of which the repayment of the oxygen deficit may only contribute partially. LaForgia *et al*. [13] reported that the post-exercise metabolic rate appears to be associated with higher lipid usage which is partially stimulated by increased catecholamine concentrations. The oxidation of lipid is known to contribute significantly to whole body energy turnover both at rest and during exercise [28,29] and it is also greatly influenced by hypoxia. As recently reported, plasma levels of malondialdehydes (MDA), as determined by thiobarbituric acid reactive substances (TBARS), increased by 56% during exercise in hypoxia compared to normoxia, suggesting a shift towards lipid substrate [30]. This may reflect a greater autonomic neuroendocrine stimulation of lipolysis during hypoxia [31,32]. In fact, plasma epinephrine and norepinephrine levels significantly increased during hypoxic exercise compared to normoxic exercise [32,33] and still elevated post-exposure [33], indicating an additive effect of hypoxia on exercise. Our outcomes revealed that passive hypoxic exposure led to a significant shift in substrate utilization towards lipid sources in PAH and PSH, respectively. Therefore passive hypoxic exposure is of sufficient amplitude to initiate acclimatory responses in sedentary people that may result in reduced body weight as previously shown in recent literature on the topic [4,5]. What effect low oxygen concentration has on post-hypoxic exposure metabolic systems related to weight loss is still not fully understood. Adaptive thermogenesis might be modified under hypobaric or normobaric hypoxia and could therefore be partially responsible for an impaired energy balance [34] and weight loss. However, the current results confirm that hypoxia modifies substrate metabolism in sedentary people and that might occur through alteration of the neuroendocrine system. For instance, adrenocorticotropic hormone (ACTH) induced steroidogenesis observed at rest and during exercise in hypoxia suggests that adrenal sensitivity for ACTH may be altered. In turn, it increases lipolytic responsiveness of adipocytes to catecholamines.

The effects of hypoxic exposure on substrate partitioning are still debated mainly because experimental designs used in the literature differ from one study to another [19]. Most of the studies that showed hypoxia induced insulin resistance and diminished glucose uptake [27,35,36] was undergone with severe hypoxic gas mixture (<10% [O_2_). However, under more moderate hypoxic exposure (>12% [O_2_), a partial pressure of oxygen corresponding to 4 000 metres of altitude and less, the metabolic response differs from the well-studied obstructive sleep apnea syndrome (OSAS) that leads to metabolic disruption [35]. For instance, Tonini *et al*. (2011) [37] observed in healthy participants a shift towards fat oxidation following 14 consecutive nights of intermittent hypoxia at 13% [O_2_. This somewhat surprising outcome was interpreted as a secondary consequence of reduced glucose uptake. According to these authors the availability of glucose or diminished glucose uptake induced by hypoxia causes the shift towards fat oxidation [38]. An alternative explanation could be that the increased sympathetic activity observed by Tamisier *et al*. (2011) [39] – same participants and same experimental design – might also explain the increased fat utilization. Repetitive severe intermittent hypoxic exposure as experienced by OSAS patients certainly leads to reduced insulin sensitivity over long periods of time [35]. However, short sessions of moderate hypoxic exposure as in the current study transiently affect fuel utilization, a positive metabolic response that resembles exercise-induced metabolic acclimation. Finally, one cannot discard that the difference between PAH and CG in the relative substrate contribution (see Table 2) might result in a distorted metabolic response owing to the very small sample size of 4 observations in the CG. However and as above-mentioned, the cumulative effect of hypoxic exposure showed that over time the substrate utilization did shift towards lipid sources to a greater magnitude compared to the acute condition and that, independently of CG metabolic response.

**Table 2:** Relative contribution of substrates

	**Acute**				**Short-term**			
	**Experimental (n = 11)**		**Control (n = 4)**		**Day one (n = 6)**		**Day Seven (n = 6)**	
	**Pre**	**Post**	**Pre**	**Post**	**Pre**	**Post**	**Pre**	**Post**
Glucose	58 ± 2	45 ± 2	38 ± 2	45 ± 3	54 ± 4	51 ± 3	44 ± 2	25 ± 3
Lipid	21 ± 1	34 ± 1	35 ± 2	31 ± 2	25 ± 4	31 ± 2	31 ± 2	46 ± 3
Protein	21 ± 1	21 ± 1	27 ± 1	23 ± 2	21 ± 1	18 ± 1	17 ± 1	19 ± 1

Mean ± SEM.

Protein oxidation rate was estimated at 66 mg·min^-1^.

### Limitation of the study

We acknowledge that the small sample size for the short-term condition might have affected the statistical power. However, we are very confident that the difference observed between the two conditions, which are at the heart of our discussion, is a true difference and not a type II error. In fact, the observed statistical power for glucose and lipid in PSH are 0.512 and 0.946, respectively. Another limitation relates to the sample size of the control group consisting of only four individuals. However, this group has been implemented to monitor for any fasting effect on the dependent variables [40,41] rather than a true control group as above-mentioned. Although the lack of biological markers related to the substrate metabolism can be viewed as a possible shortcoming, the indirect calorimetry technique has been used extensively to measure energy expenditure and substrate partitioning from respiratory gas exchanges [14,42,43] and has been proven to be a reliable and valid technique of metabolic measurement, especially at rest [44]. In addition, in this study particular caution was taken on calibration technique. Indeed, we did use the propane gas technique to calibrate the metabolic cart for very low metabolic responses. A final point that must be mentioned relates to the daily food intake log. A daily log would have provided an informative summary on participants’ food habit but data obtained from the logs were insufficient for further analyses. We are, however, confident that all participants did strictly comply with the five criteria associated with basal metabolic rate determination, a procedure that nullified the potential effect of diet.

### Potential clinical applications

If the present results are confirmed, it will lead to a new non-pharmacological strategy for the treatment of obesity. In fact, the dramatic increase in obesity worldwide being a serious threat to public health hypoxic exposure might offer a new therapeutic strategy to improve the management of weight loss. Higher aerobic pathway efficiency will lead to an increase in basal metabolic rate and daily energy expenditure. Hypoxia could, then, reverse the inefficient oxidative capacity of the obese muscle and cause weight loss.

## Conclusion

The present experiment aimed at examining the role of short-term normobaric hypoxic exposure on oxidative processes. The experimental design did evaluate and compare the efficacy of hypoxic exposures that brings global systemic physiological changes. The main results of the study confirmed that in overweight people acute and short-term normobaric hypoxia increase metabolic rate and shift substrate utilization towards lipid sources.

## Abbreviations

BMI: Body mass index; BMR: Basal metabolic rate; BP: Blood pressure; CG: Control group; CHO_ox_: Glucose oxidation; EE: Energy expenditure; FAT_ox_: Fat oxidation; HR: Heart rate; Kcal: Kilocalorie; PAH: Passive acute hypoxia; PAR-Q: Physical activity readiness questionnaire; PSH: Passive short-term hypoxia; S_p_O_2_: blood O_2_ saturation; $\dot{V}CO_{2}$: Volume of carbon dioxide; $\dot{V}O_{2}$: Volume of oxygen.

## Competing interests

The authors insure that they have no conflict of interest of any kind.

## Authors’ contributions

Both authors have equally contributed to the design of the study, the data collection and analysis, data interpretation and manuscript writing. All authors read and approved the final manuscript.
